# Beyond muscle destruction: a systematic review of rhabdomyolysis for clinical practice

**DOI:** 10.1186/s13054-016-1314-5

**Published:** 2016-06-15

**Authors:** Luis O. Chavez, Monica Leon, Sharon Einav, Joseph Varon

**Affiliations:** Universidad Autónoma de Baja California, Facultad de Medicina y Psicología, Tijuana, Baja California Mexico; Universidad Popular Autónoma del Estado de Puebla, Facultad de Medicina, Puebla, Mexico; Shaare Zedek Medical Center, Jerusalem, Israel; Hadassah-Hebrew University Faculty of Medicine, Jerusalem, Israel; Foundation Surgical Hospital of Houston TX, United States, 7501 Fannin Street, Houston, TX 77054 USA

**Keywords:** Rhabdomyolysis, Acute kidney injury, Myoglobinuria, Myopathy

## Abstract

**Background:**

Rhabdomyolysis is a clinical syndrome that comprises destruction of skeletal muscle with outflow of intracellular muscle content into the bloodstream. There is a great heterogeneity in the literature regarding definition, epidemiology, and treatment. The aim of this systematic literature review was to summarize the current state of knowledge regarding the epidemiologic data, definition, and management of rhabdomyolysis.

**Methods:**

A systematic search was conducted using the keywords “rhabdomyolysis” and “crush syndrome” covering all articles from January 2006 to December 2015 in three databases (MEDLINE, SCOPUS, and ScienceDirect). The search was divided into two steps: first, all articles that included data regarding definition, pathophysiology, and diagnosis were identified, excluding only case reports; then articles of original research with humans that reported epidemiological data (e.g., risk factors, common etiologies, and mortality) or treatment of rhabdomyolysis were identified. Information was summarized and organized based on these topics.

**Results:**

The search generated 5632 articles. After screening titles and abstracts, 164 articles were retrieved and read: 56 articles met the final inclusion criteria; 23 were reviews (narrative or systematic); 16 were original articles containing epidemiological data; and six contained treatment specifications for patients with rhabdomyolysis.

**Conclusion:**

Most studies defined rhabdomyolysis based on creatine kinase values five times above the upper limit of normal. Etiologies differ among the adult and pediatric populations and no randomized controlled trials have been done to compare intravenous fluid therapy alone versus intravenous fluid therapy with bicarbonate and/or mannitol.

## Background

Rhabdomyolysis is a clinical entity characterized by the destruction of skeletal muscle with resultant release of intracellular enzymatic content into the bloodstream that leads to systemic complications [1, 2]. The classic presentation of this condition is muscle pain, weakness, dark tea-colored urine (pigmenturia), and a marked elevation of serum creatine kinase (CK) five to ten times above the upper limit of normal serum levels [3]. The global incidence of rhabdomyolysis remains unknown but several population risk groups have been identified (i.e., morbid obese patients, chronic users of lipid-lowering drugs, post-operative patients) [4–6].

The term “crush syndrome” is usually used to describe muscle destruction after direct trauma, injury, or compression [7]. It was first described in 1941, when Bywaters and coworkers established a relationship between muscle necrosis and a brown pigment found by autopsy in the renal tubules of patients buried for several hours during a bomb attack in London [8]. Manmade and natural disasters comprise the majority of cases of crush syndrome-associated rhabdomyolysis with development of life-threatening complications to this day [7].

Acute kidney injury (AKI) is the most common systemic complication of rhabdomyolysis. It occurs at an incidence ranging between 10 and 55 % and is associated with a poor outcome, particularly in the presence of multiple organ failure [9]. Therefore, preservation of renal function with intravenous (IV) fluid therapy remains the cornerstone of rhabdomyolysis treatment [10]. The importance of rapid initiation of IV fluid therapy in the management of patients with rhabdomyolysis was first documented by Ron and coworkers in 1984 [11]; among the seven patients treated with fluids on-site during a disaster, none developed AKI. This finding received further support in additional studies suggesting that prompt IV fluid administration is associated with better patient outcome [12–14].

No guidelines for the management of rhabdomyolysis are available; nor have any randomized controlled trials of treatment been conducted. Recommendations for fluid therapy in rhabdomyolysis have yet to be established in terms of fluid type, volume, and time of initiation. Management of rhabdomyolysis is currently based on observations from retrospective studies, case reports, and case series which describe diverse and often parallel medical treatments for this syndrome and for its most common complication, AKI [10, 15].

Most of the current knowledge is based on historical data and has been unchanged for more than a decade. Therefore, the aim of this review is to summarize the literature published in the past 10 years (2006–2015) to update the definition, etiological classification, pathophysiology, diagnosis, epidemiology (e.g., risk factors, population and subgroup incidence, common etiologies, and morbidity and mortality), and treatment of rhabdomyolysis in humans.

## Methods

### Information sources

Two authors (LOC and ML) independently searched the medical literature published in three databases (MEDLINE, SCOPUS, and ScienceDirect) for articles that included in their title or abstract the keywords “rhabdomyolysis” or “crush syndrome”. The search covered all articles from January 2006 to December 2015; we selected this period to increase knowledge and provide an updated review based on the existing literature from the past 10 years. All types of articles, including reviews (narrative and systematic), randomized controlled trials (RCTs), case-control cohorts, case series, and case reports were screened for relevant content. Abstracts from the selected articles were read and, if considered eligible for further review, the complete article was obtained.

### Search approach

Data collection and extraction were divided into two steps. The first step was intended to identify the articles with data relevant for extraction regarding definition, pathophysiology, and diagnosis of rhabdomyolysis. In order to qualify for inclusion the article was to contain any information regarding the following: definition, etiological classification, pathophysiology, or diagnosis of rhabdomyolysis.

**Fig. 1:**
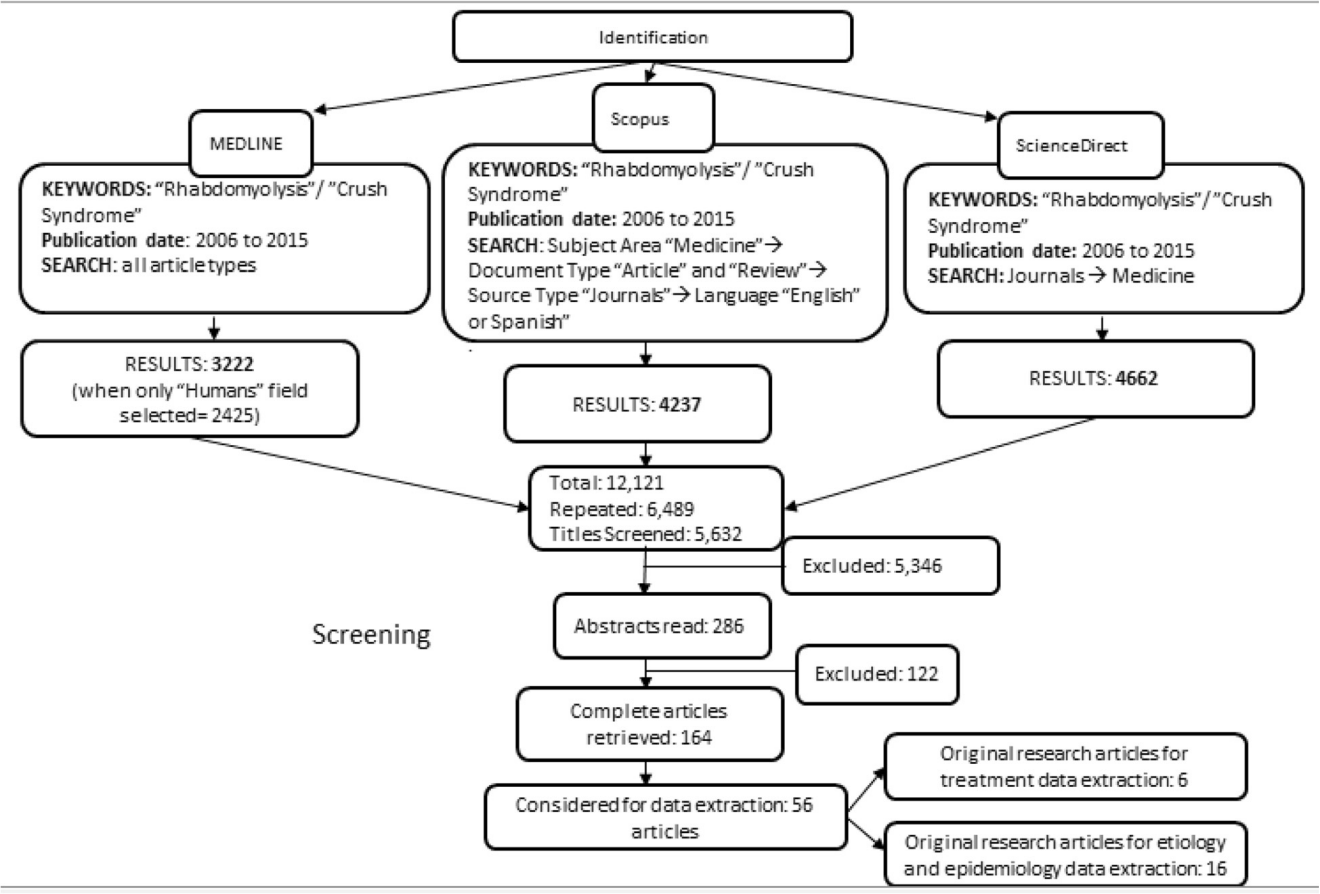
Flowchart for study selection

The second step was intended to identify original research articles that included data regarding the epidemiology (e.g., risk factors, population and subgroup incidence, common etiologies, and morbidity and mortality) or treatment of rhabdomyolysis. To this end we searched MEDLINE using the keywords noted above (“rhabdomyolysis” or “crush syndrome”) and added a filter selecting “humans” in the “Species” field. We selected for inclusion only original research articles which contained specifics of human epidemiological data or treatment. Excluded were articles referring to treatment of rhabdomyolysis-induced AKI only, case reports, and laboratory investigations of rhabdomyolysis. Repeated publications and articles not in English or Spanish were also excluded. Figure 1 shows a flowchart for study selection.

### Data collection

Controversies regarding article eligibility for data extraction were resolved by a third author (JV). The references from the selected articles that had been retrieved were also screened for additional possible references. After determining the relevance of each paper, the articles were divided into several files according to their topic relevance (definition, etiology and epidemiology, pathophysiology, diagnosis, and treatment). There was no limit to the number of files an article could appear in. Finally, the data from each topic file were summarized. No additional statistical analysis was performed.

## Results

The two searches generated 5632 articles overall. After screening the titles of all these articles, only 286 potentially relevant articles remained. The abstracts of these articles were screened and only 164 articles contained information relevant to the topic files. These 164 complete articles were retrieved and read. Only 56 articles met final inclusion criteria; single case reports and articles published in a language different from English or Spanish were excluded. This systematic review includes reference to 23 reviews (narrative or systematic) which include information regarding definition, etiological classification, pathophysiology, and diagnosis of rhabdomyolysis. It also includes reference to 16 original articles which met inclusion criteria for epidemiological data extraction and six original articles which met inclusion criteria for treatment. Table 1 lists the original articles that included data on risk factors, etiology, and epidemiology of rhabdomyolysis. Table 2 lists the original articles that included data on treatment specifications for rhabdomyolysis.

**Table 1:** Studies with epidemiological data

Article	Type of study	Type of patients	RM definition	Etiologies	Risk factors	Patients with RM	Comments
Mannix et al. 2006 [16]	RS	Pediatric patients in the ED	CK level >1000 IU/L	Viral myositis, trauma, connective tissue disease	NA	RM = 191	Most common reported symptoms were muscle pain and fever. AKI developed in only nine patients
Lagandre et al. 2006 [17]	POS	49 bariatric post-operative patients	CK level >1000 IU/L	NA	Surgical time >4 h, diabetes, BMI >40 kg/m^2^	RM = 13	Type of surgeries performed were gastric banding or bypass
De Oliveira et al. 2009 [18]	POS	22 bariatric post-operative patients	An increase >5× the upper limit of the normal CK level	NA	Prolonged surgical duration	RM = 17	Clinical neuromuscular symptoms occurred in 45 % of patients
Linares et al. 2009 [19]	RS	Hospitalized patients	CK levels >5000 IU/L	Recreational drugs and alcohol, trauma, compression, shock and statin use	NA	RM = 106	The authors suggest that RM should be defined using CK levels above 10–25 times the upper limit of normal. AKI developed in 52 patients
Youssef et al. 2010 [20]	POS	23 bariatric post-operative patients	Post-operative CK levels >1000 IU/L	NA	BMI >56 kg/m_2_	RM = 7	Factors such as sex, age, and length of surgery were not good predictors of RM
Alpers et al. 2010 [21]	RS	Patients in military training	Muscle pain, weakness, or swelling over <7 days with a CK >5× the upper limit of normal	Exertional RM	NA	RM = 177	Authors comment that exertional RM is associated with lower incidence of AKI
Bache et al. 2011 [22]	RS	76 burn patients in the ICU	“Late-onset” RM: CK >1000 U/L, 1 week or more after burn episode	NA	Sepsis, nephrotoxic drugs, hypokalemia	“Late-onset” RM = 7	Authors suggest measuring CK in all patients with the risk factors described in burn patients to initiate prompt treatment
Oshima 2011 [23]	RS	Cases of drug-related RM	NA	Drug use	<10 year olds, weight less than 50 kg	RM = 8610	Lipid lowering drugs were most frequently reported as the associated drugs
Herraez Garcia et al. 2012 [24]	RS	Adult hospitalized patients	CK level of 5× upper limit (975 UI/L)	Trauma, sepsis, immobility	Elder patients and male sex	RM = 449	No relationship was found between CK levels and AKI development or mortality
El-Abdellati et al. 2013 [25]	RS	1769 ICU patients	CK level >1000 U/L	Prolonged surgery, trauma, ischemia, infections	Surgical duration >6 h, resuscitation, compartment syndrome	RM = 342	The authors found a correlation between CK levels and the development of AKI
Rodriguez et al. 2013 [26]	RS	Acute-care hospital patients	Severe RM: >5000 IU/L	Immobilization, illicit drug abuse, infections, trauma	NA	Severe RM = 126	More than half of the patients developed AKI. Variables associated with poor outcome were hypoalbuminemia, metabolic acidosis, and decreased prothrombin time
Chen et al. 2013 [27]	RS	Pediatric patients in the ED	CK levels >1000 IU/	Infection, trauma, exercise	NA	RM = 37	Common symptoms were muscle pain and weakness. Dark urine reported in 5.4 % of patients
Talving et al. 2013 [28]	RS	Pediatric trauma patients	NA	Trauma	NA	RM = 521	AKI occurred in 70 patients. The authors concluded that a CK level ≥3000 was an independent risk factor for developing AKI
Grunau et al. 2014 [29]	RS	Patients in the ED	CK levels >1000 U/L	Illicit drug use, infections, trauma	NA	RM = 400	AKI developed in 151 patients; 18 patients required hemodialysis
van Staa et al. 2014 [30]	RS	641,703 statin users	CK levels 10× the upper limit of normal	Statin drug use	Drug–drug interaction	Reported with RM = 59 CK >10× = 182	The incidence of RM in this cohort of statin users was very low
Pariser et al. 2015 [31]	RS	1,016,074 patients with a major urologic surgery	NA	NA	Diabetes, chronic kidney disease, obesity, bleeding, age and male sex	RM = 870	Surgeries associated with RM were nephrectomy (radical or partial) and radical cystectomy

Abbreviations: *AKI* acute kidney injury, *BMI* body mass index, *CK* creatine kinase, *ED* emergency department, *ICU* intensive care unit, *NA* not available, *POS* prospective observational study, *RM* rhabdomyolysis, *RS* retrospective study

**Table 2:** Studies included with treatment details

Article	Type of study	Population	IV fluid	Bicarbonate/mannitol	Rate of AKI and need for RRT
Altintepe et al. 2007 [55]	CS	*N* = 9	Fluid type used 5 % dextrose and 0.45 NS. 4–8 L of IV fluid daily	40 mEq NaHCO_3_ and 50 mL of 20 % mannitol mixed with 1 L of IV fluid (0.45 % NaCl and 5 % dextrose) They targeted a urine pH above or equal 6.5	2 patients (28.6 %) developed AKI Patients received hemodialysis due to hyperkalemia
Cho et al. 2007 [56]	PS	*N* = 28	Fluid therapy consisted of lactated Ringer’s solution (13 patients) versus NS (15 patients) (the authors concluded that LR was more useful than NS) IV fluid rate 400 mL/h	Bicarbonate was used to achieve urine pH ≥6.5 in the patients with NS IV fluid	No patient developed AKI
Talaie et al. 2008 [51]	RS	*N* = 156	Fluid therapy given 1–8 L in the first 24 h (mean IV fluid 3.2 L/24 h)	Bicarbonate was given to 115 patients	30 patients (28.6 %) developed AKI
Zepeda-Orozco et al. 2008 [57]	RS	*N* = 28	36 % of the patients received saline infusion (20 mL/kg) in the first 24 h	79 % of patients received sodium bicarbonate IV fluid	11 patients (39.2) developed AKI 7 patients with CK levels >5000 U/L required RRT
Sanadgol et al. 2009 [58]	CS	*N* = 31	0.45 % NS	15 mEqL NaHC03 mixed with IV fluid Alkaline IV solution 3–5× more than maintenance rate was used	8 patients (25.8 %) developed AKI
Iraj et al. 2011 [34]	PS	*N* = 638	Authors recommend >6 L/day in severe RM and ≥3 L/day IV fluid in moderate RM to decrease the incidence of AKI	NA	134 patients (21 %) developed AKI 110 patients required RRT

Abbreviations: *AKI* acute kidney injury, *CS* case series, *IV* intravenous, *NA* not available, *NS* normal saline, *PS* prospective study, *RM* rhabdomyolysis, *RRT* renal replacement therapy, *RS* retrospective study

## Data synthesis

### Definition

The clinical studies were very heterogeneous with regard to the definition of rhabdomyolysis; although most authors diagnosed rhabdomyolysis based on CK levels five times the upper limit of normal levels (>1000 U/L), others used alternative criteria for diagnosis (Table 1) [16–31]. In the clinical setting, symptoms were not usually taken into consideration when defining rhabdomyolsysis; however, the most commonly included ones were muscle pain and muscle weakness while the presence of dark urine was not used to define this entity in most studies [16, 18, 21]. When rhabdomyolysis is associated with the use of lipid-lowering drugs (statins, fibrates, or a combination of both), the CK level cutoff is considered ten times the upper limit of normal [30, 32, 33]. The definition of severity of rhabdomyolysis varied among studies, some defining “severe rhabdomyolysis” based on different CK cutoff values (>5000 U/L up to >15,000 U/L) [26, 34].

### Epidemiology and etiology

Many cases of rhabdomyolysis are not detected and the incidence of this clinical entity has been reported only in subgroups of populations at risk [5, 6, 9, 35]. Rhabdomyolysis is more frequent among males, African-Americans, patients <10 and >60 years old, and in people with a body mass index exceeding 40 kg/m^2^ [5, 6].

The causes of rhabdomyolysis have been classified differently by several authors. Zimmerman and Shen [36] used a classification based on their mechanism of injury (hypoxic, physical, chemical, and biologic). Other authors described alternative classifications such as physical/non-physical, exertional/non-exertional, and acquired/inherited [2, 37–39]. Table 3 distinguishes rhabdomyolysis according to acquired and inherited causes and includes some examples of the most common etiologies reported in the medical literature. Causes of rhabdomyolysis are also different depending on the age; trauma, drugs, and infections are the most commonly reported in adults [19, 24, 26] while trauma, viral infections, drugs, and exercise etiologies prevail in children [16, 27–29].

**Table 3:** Rhabdomyolysis etiology classification [2, 25–27, 44]

Type	Cause	Examples
Acquired	Trauma	“Crush syndrome”
	Exertion	Intense muscle activity, energy depletion, electrolyte imbalance
	Ischemia	Immobilization, compression, thrombosis
	Illicit drugs	Cocaine, heroin, LSD
	Alcohol	Acute or chronic consumption
	Drugs	Dose-dependent, multiple interactions
	Infections	Bacterial, viral, parasitic
	Extreme temperatures	Hyperthermia, hypothermia, neuroleptic malignant syndrome
	Endocrinopathies	Hyper/hypo-thyroidism, diabetic complications
	Toxins	Spider bites, wasp stings, snake venom
Inherited	Metabolic myopathies	Glycogen storage, fatty acid, mitochondrial disorders
	Structural myopathies	Dystrophinopathy, dysferlinopathy
	Channel related gene mutations	RYR1 gene mutation, SCN4A gene mutation
	Others	Lipin-1 gene mutation, sickle-cell disease, “benign exertional rhabdomyolysis”

Muscle toxicity due to several drugs may arise either by a direct mechanism or through drug interactions [23, 40]. A retrospective study of 8610 cases of drug-associated rhabdomyolysis reported to the US Food and Drug Administration (FDA) from 2004 to 2009 found that simvastatin, atorvastatin, and rosuvastatin were most frequently suspected and accounted for 3945 cases (45 %) [23]. Statin drugs all have the potential to cause muscle damage in a dose-dependent manner, although they vary in several characteristics. Simvastatin, atorvastatin, and lovastatin are metabolized by CYP3A4 (the most common cytochrome P_450_ isozyme), which necessarily leads to competition with other drugs for metabolism, increasing statin blood levels and predisposing to toxicity [41]. Rosuvastatin and fluvastatin are metabolized by the CYP2A9 isozyme and therefore carry less risk of drug interaction [41]. Some degree of muscle toxicity is experienced by 0.08–10 % of patients being treated with statins alone or in combination with other lipid-lowering drugs [6, 32, 33]. However, less than 1 % have significant elevation of serum CK levels [30].

The number of rhabdomyolysis cases associated with surgery seems to have been increasing over recent years [5, 17]. Several related risk factors include extended length of surgery and comorbidities such as obesity and diabetes [17, 25, 31]. As the length of surgery increases, so does the time spent in immobility, raising the likelihood of secondary tissue compression and ischemia. Drugs used for anesthesia (propofol, barbiturates, benzodiazepines, and opiates) have also been associated with rhabdomyolysis [35].

### Pathophysiology

Regardless of the cause of rhabdomyolysis, the pathophysiology of muscle destruction follows a common pathway. The muscle cell is affected either by direct cell membrane destruction or by energy depletion [9]. Free ionized calcium enters the intracellular space and activates proteases and apoptosis pathways [2]. Production of reactive oxygen species (ROS) leads to mitochondrial dysfunction and ultimately to cell death [2, 37].

**Fig. 2:**
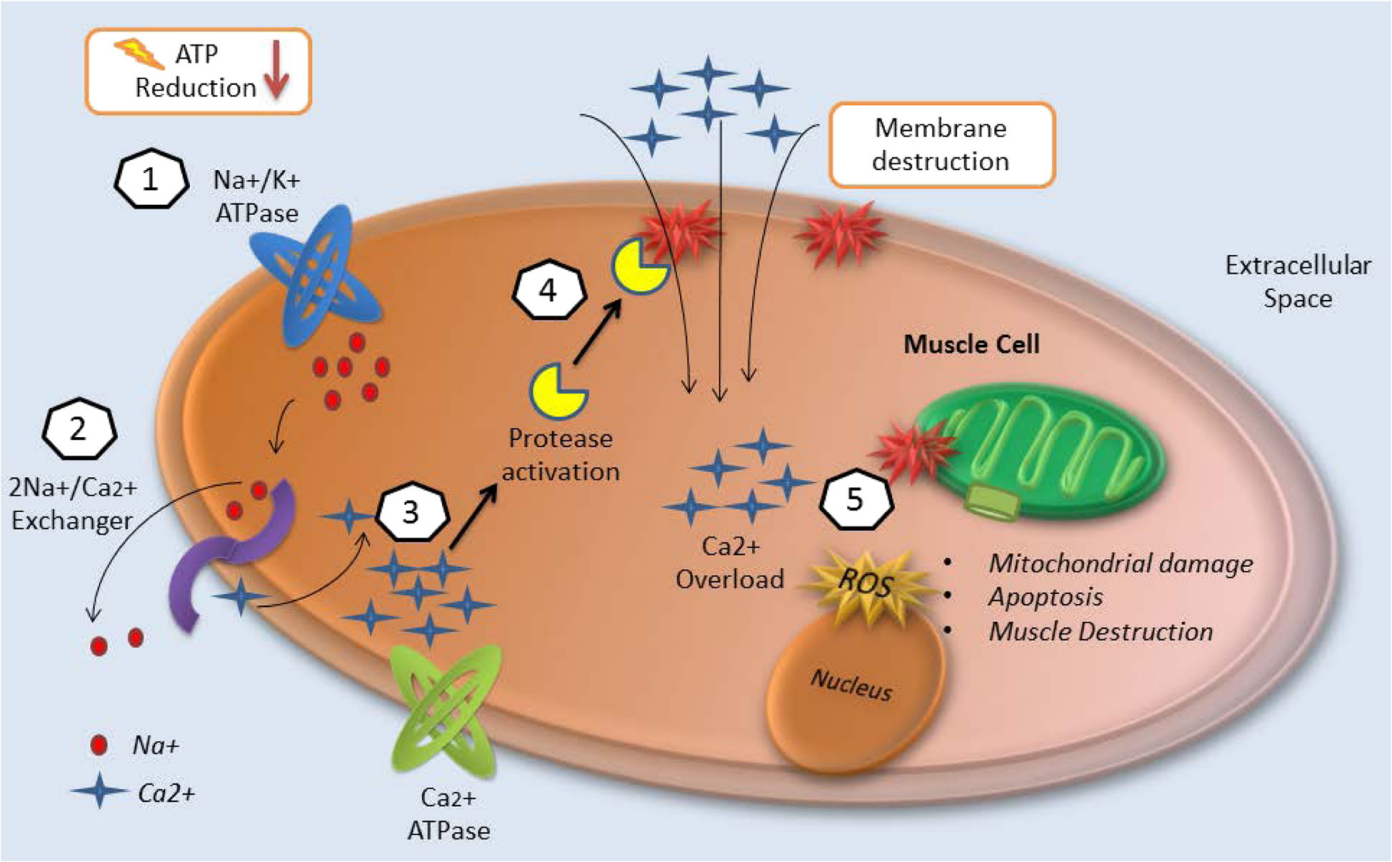
Injury mechanisms of rhabdomyolysis. (1) Energy (ATP) depletion inhibits Na^+^/K^+^ ATPase function, thus increasing intracellular sodium. (2) The 2Na^+^/Ca^2+^ exchanger increases intracellular calcium. (3) Ca^2+^ ATPase is not able to pump out intracellular calcium due to energy depletion. (4) Intracellular calcium activates proteases such as phospholipase 2 (*PLA2*), which destroy structural components of the cell membrane, allowing the entrance of more calcium. (5) Calcium overload disrupts mitochondrial integrity and induces apoptosis leading to muscle cell necrosis

Muscle cell calcium homeostasis is normally maintained by transmembrane proteins (i.e., channels, pumps), most of which are energy-dependent [42]. When energy (in the form of ATP) depletes, ATPase pump dysfunction is accompanied by an increase in intracellular Na^+^ concentration, activating the 2Na^+^/Ca^2+^ exchanger in order to correct ionic abnormalities [38]. The parallel secondary increase in intracellular calcium activates proteases such as the phospholipase A2 (PLA2) enzyme, which destroys both cellular and mitochondrial membranes [2, 37]. Figure 2 illustrates the cascade of events leading to muscle cell lysis.

Following muscle cell necrosis, release of cytotoxic intracellular components causes capillary injury and leads to third-spacing of fluids [3]. Edema, ischemia, and cell necrosis cause additional metabolic acidosis and electrolyte abnormalities, perpetuating the vicious cycle of cell death [36].

#### Mechanisms of AKI

**Fig. 3:**
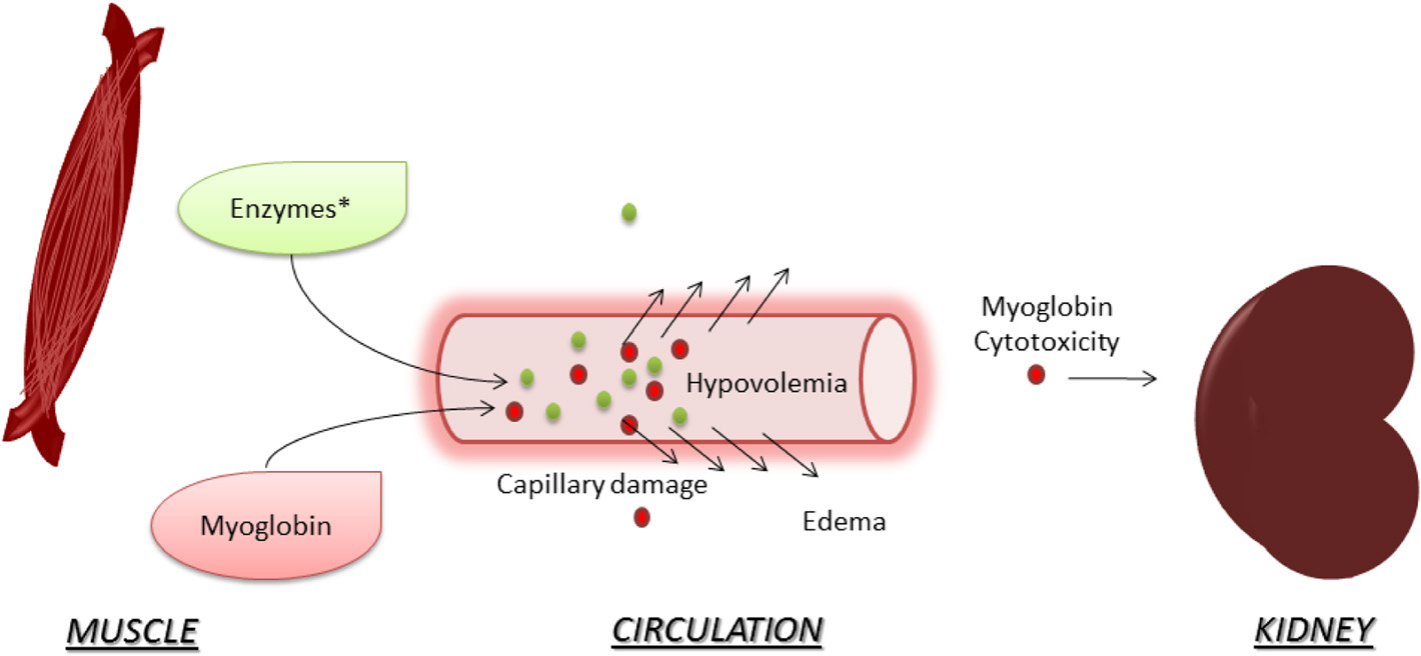
Acute kidney injury in rhabdomyolysis. Enzymes*: creatine kinase, aldolase, lactate dehydrogenase. After muscle destruction, myoglobin and enzymes released into the circulation damage capillaries, leading to leakage and edema. Hypovolemia and the decrease in renal bood flow is associated with acute kidney injury. Myoglobin cytotoxicity affects the kidney by lipid peroxidation and production of reactive oxygen species. Tubular obstruction by myoglobin is also associated with AKI

Rhabdomyolysis-associated AKI may be induced through several mechanisms, including hypovolemia, myoglobinuria, and metabolic acidosis (Fig. 3) [9, 43].

During muscle destruction, intracellular fluid is first leaked then sequestered in extracellular spaces. This depletes the intravascular volume and activates the renin–angiotensin–aldosterone system, decreasing renal blood flow [2, 9]. Release of myoglobin, the oxygen-carrier protein of the muscle, into the systemic circulation exerts a cytotoxic effect on the nephron both directly and through its compounds. The free iron released after myoglobin breakdown in the kidney reacts with hydrogen peroxide compounds (Fenton reaction), generating ROS which damage renal tubular integrity [42]. A second mechanism of kidney injury is lipid peroxidation: lipid membrane components in the kidney react with the ferryl form of myoglobin, a process called redox cycling [42]. The presence of metabolic acidosis potentiates myoglobin nephrotoxicity by promoting cast formation and tubular obstruction, particularly in the distal convoluted tubules [3].

Besides myoglobin, uric acid is also released from necrotic muscle. Uric acid forms deposits of crystals in an acidic environment, further contributing to tubular obstruction [44]. A similar pathophysiology is observed in tumor-lysis syndrome: cell damage and substance release with subsequent AKI [45].

### Diagnosis

The classic symptoms associated with rhabdomyolysis include severe muscle pain, weakness, and the presence of dark tea-colored urine, which are highly suggestive of the diagnosis [3]. Patients may also present with oliguria or even anuria [27]. Systemic circulation of intracellular muscle components can yield additional non-specific symptoms. Cardiovascular symptoms may stem from the associated electrolyte abnormalities (i.e., potassium, calcium, phosphate) and may range from cardiac dysrhythmias to cardiac arrest [10]. Patients may be hyperventilating due to pain if they are awake and agitated or hypoventilating if rhabdomyolysis was drug-induced or due to trauma [19, 28]. Drug-induced syndromes associated with rhabdomyolysis (neuroleptic malignant syndrome and malignant hyperthermia) are characterized by muscle rigidity, hyperthermia, and metabolic acidosis [10, 35].

#### Laboratory work-up

Serum CK levels gradually increase during the first 12 h of rhabdomyolysis, peak within 3–5 days, and return to baseline during the following 6–10 days [39]. Clinicians often use serum CK levels exceeding five times the upper limit of normal for diagnosing rhabdomyolysis [18, 21, 24].

Urinalysis can detect the presence of myoglobin when it exceeds 0.3 mg/L in serum [2]. The heme molecule reacts in a urine dipstick but this method cannot distinguish between a positive result due to the presence of hemoglobin or myoglobin [37]. Myoglobinuria can be considered when a patient has a reactive heme test positive for blood but the microscopic exam reveals only few red blood cells in the urine [38]. The urine pH tends to be acidic and it often contains detectable levels of protein [2, 3].

Serum CK levels have traditionally been considered the best predictor of AKI [25, 46]. However, Baeza-Trinidad and coworkers [47] recently conducted a retrospective study of 522 patients with rhabdomyolysis in which both initial CK and creatinine levels were recorded. In this study, the initial CK level was not a predictor of AKI [47]. Serum myoglobin has also been used as a predictor of AKI; Premru and coworkers [48] found that >15 mg/L of myoglobin in the blood was highly associated with development of AKI in a cohort of 484 patients. However, data regarding the use of myoglobin as an early marker of rhabdomyolysis-associated AKI remains inconclusive since many values of myoglobin overlap [49].

The Risk, Injury, Failure, Loss, and End-stage kidney disease (RIFLE) criteria are used in most studies nowadays to define AKI [50]. However, different criteria have been used to establish the diagnosis of AKI after rhabdomyolysis in clinical studies [34, 51]. Talaie and coworkers [51] diagnosed rhabdomyolysis-induced AKI in patients with a serum creatinine level elevation of more than 30 % in the first days of admission. In another study, Iraj and coworkers [34] established a diagnosis based on two repeated values of creatinine ≥1.6 mg/dL.

The presence of AKI may be accompanied by excessive potassium levels, correlating with the degree of muscle destruction. These levels should be followed closely due to the risk of cardiac dysrhythmias [36]. Serial electrocardiography studies should also be performed to detect abnormalities secondary to electrolyte disturbances [10].

Prolongation of the prothrombin time, thrombocytopenia, and high levels of fibrinogen degradation products may also be detected during rhabdomyolysis [10, 52]. In this setting, serial blood tests are indicated to detect disseminated intravascular coagulopathy as early as possible. Arterial blood gases typically demonstrate metabolic acidosis with an elevated anion gap, reflecting the increase in organic acid levels in the serum due to muscle necrosis [2, 26].

Initial CK and myoglobin levels are inconsistent in predicting mortality or AKI in rhabdomyolysis [49, 53]. McMahon and coworkers [53] have recently validated an instrument for predicting mortality and AKI. This score includes eight variables: age, gender, etiology, and initial levels of creatinine, calcium, phosphate, and serum bicarbonate.

### Treatment

Treatment of the underlying source of muscle injury, once identified, is the first component of successful management. This may include cessation of a potentially harmful drug, control of patient temperature, treatment of underlying infection, and more [7, 51].

#### IV fluid therapy

Fluid replacement is the keystone of rhabdomyolysis treatment. Capillary damage and fluid leakage lead to a “functional” dehydration that requires rapid correction [54]. Early, aggressive fluid therapy increases renal blood flow, thereby increasing secretion of nephrotoxic compounds that may cause AKI [9].

Table 2 shows studies in which IV fluid therapy was described for patients diagnosed with rhabdomyolysis [34, 51, 55–58]. The type of IV fluid varied from the combination of 5 % dextrose and 0.45 normal saline (NS), lactated Ringer’s solution, and NS solution with or without bicarbonate [51, 55, 56]. Fluid administration was reported either as an hourly or daily rate. Cho and coworkers [56] prospectively studied 28 patients treated with either NS or lactated Ringer’s solution with an IV fluid rate of 400 mL/h and none of the patients developed AKI. Other studies used from 4 to 8 L of IV fluid daily [34, 51, 55].

In 2013, Scharman and coworkers [54] conducted a systematic review of therapies for prevention of rhabdomyolysis-associated AKI; overall, 27 studies were included. The authors concluded that IV fluid therapy should ideally be initiated within 6 h of muscle injury, targeting a urine output of 300 mL/h. No specific recommendations were provided regarding the type of fluid because of the variety of intravenous fluids used in the different studies [54].

In non-traumatic rhabdomyolysis, the use of lactated Ringer’s solution was compared with NS in a cohort of 28 patients divided into 13 patients treated with Ringer’s solution and 15 patients treated with NS. No significant difference was found either in the rate of reduction of CK levels or in the prevalence of AKI in both groups [56]. Despite the poor literature comparing different fluids, Sever and coworkers suggested in a supplement published in *Nephrology, Dialysis, Transplantation* entitled “Recommendation for the management of crush victims in mass disasters” that isotonic saline should be the initial choice for volume correction in rhabdomyolysis secondary to crush injury [15]. These authors also suggested that initial fluid infusion rates should be 1 L/h for 2 h after injury and 500 mL/h after 120 minutes [15]. However, these recommendations were not based on randomized clinical trials. Patients receiving fluid replacement therapy should be monitored closely to prevent complications such as fluid overload and metabolic acidosis [59].

#### Treatment of electrolyte abnormalities

Reinstatement of the biochemical equilibrium during rhabdomyolysis should be undertaken with care in order to avoid adverse effects of treatment. Hyperkalemia is the only electrolyte abnormality that requires rapid correction in order to reduce the risk of cardiac dysrhythmias [43, 60]. Administration of calcium chloride/gluconate for hypocalcemia should be avoided since calcium supplementation may increase muscle injury [10]. Correction of hyperphosphatemia requires careful monitoring of both phosphorus and calcium levels since increased levels of phosphorus may promote calcium deposition in necrotic muscle tissue [10].

#### Bicarbonate for prevention of AKI

The use of bicarbonate for prevention of AKI is based on the concept that an acidic environment promotes myoglobin toxicity; hence, an alkali urine environment may reduce redox-cycling, lipid peroxidation, and myoglobin cast formation [9]. It is thus plausible that increasing urine pH above 6.5 with intravenous sodium bicarbonate could prevent AKI [55]. Besides AKI prevention, several authors have suggested that sodium bicarbonate should be used to correct metabolic acidosis [38]. However, administration of sodium bicarbonate may also produce paradoxical intracellular acidosis and volume overload, particularly in patients with respiratory or circulatory failure, when the bicarbonate buffering system shifts to increase circulating carbon dioxide (HCO_3_ + H^+^ → H_2_CO_3_ → H_2_0 + CO_2_) [61].

Table 2 includes some of the “bicarbonate cocktails” added to IV fluid therapy in some studies. However, none of the studies have actually compared this therapy with intravenous fluid therapy alone [51, 58].

#### Mannitol

There is no consensus regarding the use of mannitol since its side effects include volume depletion and potentially worsening pre-renal azotemia [9]. However, the theoretical benefits of mannitol administration include improved diuresis, increased renal perfusion, excretion of myoglobin, and a direct antioxidant effect on renal parenchyma [62]. Authors that recommend using mannitol suggest it should only be administered if fluid therapy alone does not lead to urine output exceeding 300 mL/h [15]. Mannitol should be avoided in anuric patients; it is therefore recommended to assess the urinary response starting only with IV fluids prior to deciding whether to proceed with mannitol administration [15].

#### Continuous renal replacement therapy

Continuous renal replacement therapy (CRRT) clears myoglobin from the bloodstream, thereby potentially decreasing the amount of renal damage [63, 64]. Zeng and coworkers [60] systematically reviewed the potential benefits of CRRT in patients with rhabdomyolysis and AKI. The authors found only three studies for inclusion in their review (*n* = 101 patients). They concluded that, despite the improvement in myoglobin, creatinine, and electrolyte levels in patients treated with CRRT, mortality rates remained unchanged [60]. CRRT should therefore only be considered when life-threatening electrolyte abnormalities emerge as complications of AKI that are non-responsive to initial therapy [43].

## Conclusions

Rhabdomyolysis remains a major clinical challenge. Non-specific symptoms, multiple etiologies, and systemic complications obscure the diagnosis and complicate the treatment of this condition. The pathophysiology of myoglobin-induced injury to the renal parenchyma has been elucidated and aggressive fluid therapy remains the keystone of therapy. However, RCTs are sorely lacking regarding the use of both fluids and adjuvant pharmacological therapies (mannitol and bicarbonate) for AKI prevention. CRRT improves myoglobin clearance but does not change mortality. Several important aspects of rhabdomyolysis should be addressed in the future: a homogenous definition should be created for this syndrome, data from past cases should be pooled to derive and validate the best marker for predicting development of AKI, and multicenter RCTs that compare standardized intravenous fluid therapy alone with fluids with sodium bicarbonate and/or mannitol should be planned.

## Abbreviations

AKI: acute kidney injury
CK: creatine kinase
CRRT: continuous renal replacement therapy
IV: intravenous
NS: normal saline
RCT: randomized controlled trial
ROS: reactive oxygen species

## References

[1] Huerta-Alardin AL, Varon J, Marik PE (2005). Bench-to-bedside review: rhabdomyolysis—an overview for clinicians. Crit Care.

[2] Giannoglou GD, Chatzizisis YS, Misirli G (2007). The syndrome of rhabdomyolysis: pathophysiology and diagnosis. Eur J Intern Med.

[3] Bagley WH, Yang H, Shah KH (2007). Rhabdomyolysis. Intern Emerg Med..

[4] Landau ME, Kenney K, Deuster P, Campbell W (2012). Exertional rhabdomyolysis: a clinical review with a focus on genetic influences. J Clin Neuromuscul Dis.

[5] Chakravartty S, Sarma DR, Patel AG (2013). Rhabdomyolysis in bariatric surgery: a systematic review. Obes Surg.

[6] Iwere RB, Hewitt J (2015). Myopathy in older people receiving statin therapy: a systematic review and meta-analysis. Br J Clin Pharmacol.

[7] Mehmet SS, Lameire N, Biesen WV, Vanholder R (2015). Disaster nephrology: a new concept for an old problem. Clin Kidney J..

[8] Bywaters E, Beall D (1941). Crush injuries with impairment of renal function. Br Med J..

[9] Bosch X, Poch E, Grau JM (2009). Rhabdomyolysis and acute kidney injury. N Engl J Med.

[10] Chatzizisis YS, Misirli G, Hatzitolios AI, Giannoglou GD (2008). The syndrome of rhabdomyolysis: complications and treatment. Eur J Intern Med.

[11] Ron D, Taitelman U, Michaelson M, Bar-Joseph G, Bursztein S, Better OS (1984). Prevention of acute renal failure in traumatic rhabdomyolysis. Arch Intern Med.

[12] Sheng ZY (1987). Medical support in the Tangshan earthquake: a review of the management of mass casualties and certain major injuries. J Trauma..

[13] Brown C, Rhee P, Chan L, Evans K, Demetriades D, Velmahos GC (2004). Preventing renal failure in patients with rhabdomyolysis: do bicarbonate and mannitol make a difference?. J Trauma.

[14] Melli G, Chaudhry V, Cornblath DR (2005). Rhabdomyolysis: an evaluation of 475 hospitalized patients. Medicine (Baltimore).

[15] Sever MS, Vanholder R, Ashkenazi L, Becker G, Better O, Covic A (2012). Recommendation for the management of crush victims in mass disasters. Nephrol Dial Transplant..

[16] Mannix R, Tan ML, Wright R, Baskin M (2006). Acute pediatric rhabdomyolysis: causes and rates of renal failure. Pediatrics..

[17] Lagandre S, Arnalsteen L, Vallet B, Robin E, Jany T, Onraed B (2006). Predictive factors for rhabdomyolysis after bariatric surgery. Obes Surg.

[18] de Oliveira LD, Diniz MT, de Fatima HS, Diniz M, Savassi-Rocha AL, Camargos ST (2009). Rhabdomyolysis after bariatric surgery by Roux-en-Y gastric bypass: a prospective study. Obes Surg.

[19] Linares LA, Golomb BA, Jaojoco JA, Sikand H, Phillips PS (2009). The modern spectrum of rhabdomyolysis: drug toxicity revealed by creatine kinase screening. Curr Drug Saf.

[20] Youssef T, Abd-Elaal L, Zakaria G, Haasheesh M (2010). Bariatric surgery: rhabdomyolysis after open Roux-en-Y gastric bypass: a prospective study. Int J Surg.

[21] Alpers JP, Jones LK (2010). Natural history of exertional rhabdomyolysis: a population-based analysis. Muscle Nerve.

[22] Bache SE, Taggart I, Gilhooly C (2011). Late-onset rhabdomyolysis in burn patients in the intensive care unit. Burns.

[23] Oshima Y (2011). Characteristics of drugs-associated rhabdomyolysis: analysis of 8,610 cases reported to the U.S. Food and Drug administration. Intern Med.

[24] Herraez Garcia J, Torracchi Carrasco AM, Antoli-Royo AC (2012). Rhabdomyolysis: a descriptive study of 449 patients. Med Clin (Barc).

[25] El-Abdellati E, Eyselbergs M, Sirimsi H, Hoof VV, Wouters K, Verbrugghe W (2013). An observational study on rhabdomyolysis in the intensive care unit. Exploring its risk factors and main complication: acute kidney injury. Ann Intensive Care..

[26] Rodriguez E, Soler MJ, Rap O, Barrios C, Orfila MA, Pascual J (2013). Risk factors for acute kidney injury in severe rhabdomyolysis. PLoS One.

[27] Chen CY, Lin YR, Zhao LL, Yang WC, Chang YJ, Wu KH (2013). Clinical Spectrum of rhabdomyolysis presented to pediatric emergency department. BMC Pediatr.

[28] Talving P, Karaanos E, Skiada D, Lam L, Teixeira P, Inaba K (2013). Relationship of creatine kinase elevation and acute kidney injury in pediatric trauma patients. J Trauma Acute Care Surg.

[29] Grunau BE, Pourvali R, Wiens MO, Levin A, Li J, Grafstein E (2014). Characteritics and thirty-day outcomes of emergency department patients with elevated creatine kinase. Acad Emerg Med.

[30] van Staa TP, Carr DF, O’Meara H, McCann G, Pirmohamed M (2014). Predictors and outcomes of increases in creatine phosphokinase concentrations or rhabdomyolysis risk during statin treatment. Br J Clin Pharmacol.

[31] Pariser JJ, Pearce SM, Patel SG, Anderson BB, Packiam VT, Shalhav AL (2015). Rhabdomyolysis after major urologic surgery: epidemiology, risk factors, and outcomes. Urology.

[32] Antons KA, Williams CD, Baker SK, Phillips PS (2006). Clinical perspectives of statin-induced rhabdomyolysis. Am J Med.

[33] Harper CR, Jacobson TA (2007). The broad spectrum of statin myopathy: from myalgia to rhabdomyolysis. Curr Opin Lipidol.

[34] Iraj N, Saeed S, Mostafa H, Houshang S, Ali S, Farin RF (2011). Prophylactic fluid therapy in crushed victims of Bam earthquake. Am J Emerg Med..

[35] Hohenegger M (2012). Drug induced rhabdomyolysis. Curr Opin Pharmacol.

[36] Zimmerman JL, Shen MC (2013). Rhabdomyolysis. Chest..

[37] Torres PA, Helmestetter JA, Kaye AM, Kaye AD (2014). Rhabdomyolysis: pathogenesis, diagnosis, and treatment. Ochsner J.

[38] Zutt R, van der Kooi AJ, Linthorst GE, Wanders RJ, de Visser M (2014). Rhabdomyolysis: review of the literature. Neuromuscul Disord.

[39] Nance JR, Mammen AL (2015). Diagnostic evaluation of rhabdomyolysis. Muscle Nerve.

[40] Prendergast BD, George CF (1993). Drug-induced rhabdomyolysis: mechanisms and management. Postgrad Med J..

[41] Norata GD, Tibolla G, Catapano AL (2014). Statins and skeletal muscles toxicity: from clinical trials to everyday practice. Pharmacol Res..

[42] Boutaud O, Roberts LJ (2011). Mechanism-based therapeutic approaches to rhabdomyolysis-induced renal failure. Free Radic Biol Med.

[43] Petejova N, Martinek A (2014). Acute kidney injury due to rhabdomyolysis and renal replacement therapy: a critical review. Crit Care.

[44] Shimada M, Dass B, Ejaz AA (2011). Paradigm shift in the role of uric acid in acute kidney injury. Semin Nephrol.

[45] Firwana BM, Hasan R, Hasan N, Alahdab F, Alnahhas I, Hasan S (2012). Tumor lysis syndrome: a systematic review of case series and case reports. Postgrad Med.

[46] de Meijer AR, Fikkers BG, de Keijzer MH, van Engelen BG, Drenth JP (2003). Serum creatine kinase as predictor of clinical course in rhabdomyolysis a 5-year intensive care survey. Intensive Care Med.

[47] Baeza-Trinidad R, Brea-Hernando A, Morera-Rodriguez S, Brito-Diaz Y, Sanchez-Hernandez S, El Bikri L (2015). Creatinine as predictor value of mortality and acute kidney injury in rhabdomyolysis. Intern Med J.

[48] Premru V, Kovač J, Ponikvar R (2013). Use of myoglobin as a marker and predictor in myoglobinuric acute kidney injury. Ther Apher Dial..

[49] Rodriguez-Capote K, Balion CM, Hill SA, Cleve R, Yang L, El Sharif A (2009). Utility of urine myoglobin for the prediction of acute renal failure in patients with suspected rhabdomyolysis: a systematic review. Clin Chem.

[50] Thomas ME, Blaine C, Dawnay A, Devonald MA, Ftouh S, Laing C (2015). The definition of acute kidney injury and its use in practice. Kidney Int.

[51] Talaie H, Emam-Hadi M, Panahandeh R, Hassanian-Moghaddam H, Abdollahi M (2008). On the mechanisms underlying poisoning-induced rhabdomyolysis and acute renal failure. Toxicol Mech Methods..

[52] Cervellin G, COmelli I, Lippi G (2010). Rhabdomolysis: historical background, clinical, diagnostic and therapeutic features. Clin Chem Lab Med.

[53] McMahon GM, Zeng X, Waikar SS (2013). A risk prediction score for kidney failure or mortality in rhabdomyolysis. JAMA Intern Med.

[54] Scharman EJ, Troutman WG (2013). Prevention of kidney injury following rhabdomyolysis: a systematic review. Ann Pharmacother.

[55] Altintepe L, Guney I, Tonbul Z, Turk S, Mazi M, Agca E (2007). Early and intensive fluid replacement prevents acute renal failure in the crush cases associated with spontaneous collapse of an apartment in Konya. Ren Fail.

[56] Cho YS, Lim H, Kim SH (2007). Comparison of lactated Ringer’s solution and 0.9 % saline in the treatment of rhabdomyoysis induced by doxylamine intoxication. Emerg Med J.

[57] Zepeda-Orozco D, Ault BH, Jones DP (2008). Factors associated with acute renal failure in children with rhabdomyolysis. Pedatr Nephrol..

[58] Sanadgol H, Najafi I, Rajabi Vahid M, Hossini M, Ghafari A (2009). Fluid therapy in pediatric victims of the 2003 Bam, Iran earthquake. Prehosp Disaster Med..

[59] Myburgh JA, Mythen MG (2013). Resuscitation fluids. N Engl J Med..

[60] Zeng X, Zhang L, Wu T, Fu P (2014). Continuous renal replacement therapy (CRRT) for rhabdomyolysis. Cochrane Database Syst Rev..

[61] Berend K, de Vries AP, Gans RO (2015). Physiological approach to assessment of acid-base disturbances. N Engl J Med.

[62] Bragadottir G, Redfors B, Ricksten SE (2012). Mannitol increases renal blood flow and maintains filtration fraction and oxygenation in postoperative acute kidney injury: a prospective interventional study. Crit Care..

[63] Sorrentino SA, Kielstein JT, Lukasz A, Sorrentino JN, Gohrbandt B, Haller H (2011). High permeability dialysis membrane allows effective removal of myoglobin in acute kidney injury resulting from rhabdomyolysis. Crit Care Med..

[64] Heyne N, Guthoff M, Krieger J, Haap M, Häring HU (2012). High cut-off renal replacement therapy for removal of myoglobin in severe rhabdomyolysis and acute kidney injury: a case series. Nephron Clin Pract..

